# Interactional synchrony: signals, mechanisms and benefits

**DOI:** 10.1093/scan/nsaa024

**Published:** 2020-03-03

**Authors:** Stefanie Hoehl, Merle Fairhurst, Annett Schirmer

**Affiliations:** Department of Developmental and Educational Psychology, Faculty of Psychology, University of Vienna, Liebiggasse 5, 1010 Vienna, Austria; Institute for Psychology, Bundeswehr University Munich, Germany; Munich Center for Neuroscience, Ludwig Maximilian University, Germany; Department of Psychology, The Chinese University of Hong Kong, 3rd Floor, Sino Building, Shatin, N.T., Hong Kong; Brain and Mind Institute, The Chinese University of Hong Kong, 3rd Floor, Sino Building, Shatin, N.T., Hong Kong; Center for Cognition and Brain Studies, The Chinese University of Hong Kong, 3rd Floor, Sino Building, Shatin, N.T., Hong Kong

**Keywords:** interactional rhythm, entrainment, timing, social bonding

## Abstract

Many group-living animals, humans included, occasionally synchronize their behavior with that of conspecifics. Social psychology and neuroscience have attempted to explain this phenomenon. Here we sought to integrate results around three themes: the stimuli, the mechanisms and the benefits of interactional synchrony. As regards stimuli, we asked what characteristics, apart from temporal regularity, prompt synchronization and found that stimulus modality and complexity are important. The high temporal resolution of the auditory system and the relevance of socio-emotional information endow auditory, multimodal, emotional and somewhat variable and adaptive sequences with particular synchronizing power. Looking at the mechanisms revealed that traditional perspectives emphasizing beat-based representations of others’ signals conflict with more recent work investigating the perception of temporal regularity. Timing processes supported by striato-cortical loops represent any kind of repetitive interval sequence fairly automatically. Additionally, socio-emotional processes supported by posterior superior temporal cortex help endow such sequences with value motivating the extent of synchronizing. Synchronizing benefits arise from an increased predictability of incoming signals and include many positive outcomes ranging from basic information processing at the individual level to the bonding of dyads and larger groups.

## Introduction

Sitting at the piano, our friend and foe, the metronome, ticks and waves. How hard can it be—keeping in time, not playing too fast or too slow? The answer is ‘incredibly hard’ as humans vary naturally when trying to align their movements with an external rhythm (Dahan *et al.*, 2016; Mills *et al.*, 2019). Yet, our performance error is small if not negligible when compared with that of our primate relatives and many other species attempting a similar task (for a review, see Wilson and Cook, 2016). Although most group-living animals show some form of synchronizing when moving in a shoal, flock or herd, humans have taken this behavior several steps forward (for a review, see Schirmer *et al.*, 2016b). We not only align frequently and effortlessly with each other, we also spontaneously and persistently converge our behavior to fairly artificial stimuli like a metronome.

Synchronizing with others and with musical rhythms is fun. We seek it out when going to the gym, attending a dance class or singing in a choir (Weinstein *et al.*, 2016). Moreover, synchronizing seemingly diffuses through the body. Rather than being restricted to overt motor acts like singing, tapping or dancing, it occurs at multiple levels. We see it in the coupling of cardiac activity between a mother and her unborn child (Feldman, 2007), in concurrent changes in pupil size between parents and their infants (Fawcett *et al.*, 2017) or in the alignment of oscillations in neuronal polarization between two individuals talking to each other (Pérez *et al.*, 2017). As such, synchronizing is a fairly complex phenomenon that, although much investigated, is still poorly understood.

Here, we sought to address this situation by reviewing a broad range of articles on rhythmic processes examining human and non-human data, looking at various forms of measurement and tackling functions as diverse as finger tapping, visual target detection, drumming, emotion regulation and whacking a mole. Our goal was to integrate disparate approaches and findings and to develop a theoretical perspective that can guide future attempts at understanding the synchronizing process.

In pursuit of this goal, we organized this article around three themes. First, we addressed the characteristics of stimuli with high synchronizing power as compared to low synchronizing power. Although simple sounds like those coming from a metronome guide the timing of music-making, not all sounds or objects have the same potential to make us swing along. Second, we summarized results pointing to the mechanisms that underpin interactional synchrony. In doing so, we established important links to broader mental functions associated with stimulus regularity and prediction. Lastly, we considered the consequences responsible for the pervasiveness of synchronicity in our social lives. Specifically, we asked how synchronizing benefits us as individuals and as a primate species.

## What makes us synchronize?

Signals that vary at regular intervals can influence the timing of an observer’s internal and external processes. But what, apart from their temporal regularity, determines their synchronizing effect? In this section, we will consider this question paying particular attention to (i) the role of signal modality, (ii) the signal’s emotional relevance and (iii) potential differences between signals from a real to a virtual agent. In pursuing these points, we focus on different aspects of signal complexity, thus enabling insights into the synchronizing power of relatively simple as compared to multidimensional stimuli. Moreover, we suggest that complex feature extraction plays an important part in prompting synchronization and thus facilitating the coordination of behaviors in many group-living animals, a process that is of particular relevance to humans (Launay *et al.*, 2016).

### Signal modality

It is widely reported that behavioral synchronization performance is modality–dependent. Temporal alignment accuracy is lowest for auditory relative to other signals. Evidence for this comes from finger–tapping studies that examined tapping asynchrony, which is the temporal delay between taps and a pacing signal, and tapping consistency, which is the variability of tap-to-tap intervals. Tapping asynchrony is lowest and tapping consistency highest for auditory, followed by tactile and then visual stimuli (Repp and Su, 2013).

Attempts to explain these differences have centered on whether and how the modalities should be compared. In this context, it has been suggested that perhaps our natural environment entails fewer visual than auditory rhythms leading to differences in exposure and processing practice (Varlet *et al.*, 2012). However, whether such differences exist is questionable. Indeed, visual rhythms abound whether it is in the form of a tree waving, a cursor blinking or a person’s eyelids opening and closing. Also, as objects must move to create sound, they often engender temporally coupled auditory and visual oscillations as in when birds flap their wings or horses move on hard ground.

In light of this, the stimulus compatibility account has been proposed as an alternative explanation of modality effects (Hove *et al.*, 2013). This account holds that synchronization depends on how stimulus properties align with modality-specific sensitivities (e.g. Schirmer, 2018). For example, basic perceptual research showed that audition and vision treat spatio-temporal stimulus features in different ways (Mahar *et al.*, 1994). While audition emphasizes temporal analysis, vision emphasizes spatial analysis. Thus, visual signals moving across space synchronize motor output more efficiently than static visual signals and reduce (but not eliminate) the performance gap to the auditory modality (Hove *et al.*, 2013).

Although unimodal stimuli have some ecological relevance, everyday perceptual experiences are often more complex engaging multiple modalities concurrently. Research has demonstrated that multimodal integration depends on the relation between signal and noise (Ernst and Banks, 2002) as well as on the temporal regularity of individual modality streams (Elliott *et al.*, 2010). Features of these streams will determine whether and how the information they provide informs behavior. For example, in a badly edited video with lags between vision and audition, synchronization occurs to the modality that offers a better signal-to-noise ratio and greater temporal regularity (Bishop and Goebl, 2015).

Even in a supposedly unimodal synchronization task, multisensory integration emerges from self-related feedback associated with the performed action. When tapping a finger, stamping a foot or waving an arm in time with a pacing stimulus, movement produces a sensory event. This event may be felt, heard or seen and thus modulate synchronization performance. In general such self-related feedback facilitates and its absence impairs synchrony (Aschersleben *et al.*, 2001; Goebl and Palmer, 2008; Maidhof *et al.*, 2013). New research geared toward rehabilitation and training has compared visual–tactile, visual–audio, visual–audio–tactile and visual-only feedback. Their results show comparable motion accuracy across all conditions but smoother movements for visual–tactile than visual–audio feedback (Feng and Stockman, 2019).

Data suggest that the degree of synchrony arising from multisensory input depends on what modalities are involved. If one modality is auditory, it will bias temporal processing even if it is fairly noisy or irregular (Elliott *et al.*, 2010). Nevertheless, multisensory cues generally facilitate synchronization with bimodal events producing better performance than unimodal ones (Armstrong and Issartel, 2014). As ever, context is key and divergent effects of multisensory processing may depend on the stimulus as it is embedded in the task.

Verbal in-person interactions presents a special case of multisensory integration in which temporal features are extracted from, for example, the sound of spoken words, lip movements and additional gestures of the head and hands (Munhall *et al.*, 2004). Although in this modern age, there are counter examples in which we rely on an isolated visual (email) or auditory (phone) channel, face-to-face communication remains a crucial element of our lives with privileged processing mechanisms that benefit from synchronization (Amiriparian *et al.*, 2019) via temporally coupled neural responses to frequency-specific features in the signals (Jiang *et al.*, 2012). Possibly such neuronal synchronization supports goal-relevant behavioral coordination, including the mimicry of grammatical forms (Hasson *et al.*, 2012).

In summary, many events in our environment afford an opportunity to synchronize. Some events, such as voices in another room, are perceived unimodally, whereas many others engage multimodal processing. Both unimodal and multimodal signals facilitate synchronizing especially when they include auditory information likely because the auditory system is particularly suited for representing time the most critical information in the context of temporal coordination. Although unimodal streams enable us to synchronize, they do so less efficiently than multimodal streams.

### Signal emotion

Musical stimuli that are perceived as ‘activating’ or ‘relaxing’ prompt different walking speeds despite having the same tempo (Leman *et al.*, 2013). This suggests that, apart from modality, other stimulus features are relevant. Specifically, those prompting some sort of emotional response might contribute to our propensity to synchronize.

Indeed, a role for signal emotion has been demonstrated at different synchronizing levels. Behaviorally, emotions affect the readiness with which individuals temporally align. Positive emotions enhance, whereas negative emotions impede alignment. This has been demonstrated with a range of paradigms including passive music listening with visual targets occurring on weak and strong musical beats. Responses at both beats were facilitated by consonant relative to dissonant music (Trost *et al.*, 2014). At a physiological level, happy and sad music differently excite the autonomic nervous system. The former arouses listeners more strongly than the latter, thus augmenting entrainment to fast temporal rhythms (Khalfa *et al.*, 2008). At a neural level, positive entraining stimuli activate brain regions relevant for attention (Trost *et al.*, 2014). Additionally, highly arousing musical excerpts are particularly suited to evoke neural activation that is shared among listeners in key emotion areas such as the amygdala, insula and caudate nucleus (Trost *et al.*, 2015). While amygdala activation seems related to musical energy (e.g. intensity and dissonance), insula activation appears to be coupled to acoustic event density. Together, these effects suggest that expressive or emotional signals enhance the brain representation of associated temporal features and facilitate behavioral change with potential feedback on emotion processing in, for example, reward circuitry (e.g. Schilbach *et al.*, 2010; for a review see Launay *et al.*, 2016).

It remains debated how emotions influence synchronizing. One possibility is that their influence is only indirect through changes in timing (Repp, 2002). Simple timing tasks such as judging stimulus duration or comparing two intervals elicit performance differences as a function of stimulus emotion (for a review, see Droit-Volet and Meck, 2007; Lake *et al.*, 2016; Schirmer, 2016). For example, angry faces are typically perceived as temporally longer than same-duration neutral faces (Gil and Droit-Volet, 2011; Fayolle and Droit-Volet, 2014). This effect has been attributed to arousal increasing the speed of an internal clock mechanism (Droit-Volet and Meck, 2007). Additionally, it has been explained in reference to the relation between emotion and attention. Emotional stimuli attract greater attention than neutral stimuli, facilitating the accumulation of perceptual time (Lui *et al.*, 2011).

Yet, apart from having an indirect effect, emotions may shape synchronizing directly by motivating general stimulus processing and the alignment of internal with external rhythms. Accordingly, there is a positivity bias in synchronizing, whereas effects of emotions on timing show typically irrespective of valence. Moreover, positive emotions broaden attention and enhance flexibility, whereas negative emotions have the opposite effect (Fredrickson, 1998) including in the auditory domain (Putkinen *et al.*, 2017). Thus, we entertain the possibility that emotions, especially when they are positive, prepare individuals for and facilitate the representation of another’s thoughts, feelings and behaviors including their temporal organization (Keysers *et al.*, 2010; Nummenmaa *et al.*, 2012).

### Real *vs* virtual agents

Most synchronization research either examines human dyads or looks at interactions between a human and an artificial agent. To date, only few studies have attempted to compare the two. Kirschner and Tomasello (2009) examined the joint drumming of preschool children showing that drumming accuracy was higher when the drumming partner was a human as compared to a computer. Although more complex in nature, an influence of partner type was also demonstrated in adults (Mills *et al.*, 2019).

In light of these findings, what are ‘human-like’ aspects of the stimulus that modulate synchronization? Work by Dahan *et al.* (2016) points to the importance of a random motion element. Specifically, they modeled synchronizing to a pacing signal by including a model term that increases with ongoing zero-phase synchrony and when crossing some threshold calls for a random force that allows the model to exit from synchrony. Compared to models without such a term, this model produces signal tracking performance with similar temporal variation as found in human data.

Additionally, Washburn *et al.* (2019) recently demonstrated the importance of feedback delays. Feedback delays concern the time that lapses between an input signal, its perception, the perceiver’s motor response and the perception of the associated response consequences (e.g. tactile, auditory, visual). Traditionally, such delays were thought to impair performance. However, more recently it was shown that they can be useful by increasing adaptivity. When introduced to an artificial agent, a small feedback delay enhances the agent’s ability to anticipate chaotic human behavior but also, and perhaps more importantly, to synchronize with such behavior in a manner similar to natural human–human anticipatory synchronization (Washburn *et al.*, 2019).

### Stimulus complexity powers synchrony

Although a simple rhythmical stimulus such as an isochronously repeated tone can elicit synchronization, more complex stimuli can be more powerful in prompting us to temporally align. The present survey of different stimulus dimensions revealed benefits for moving as compared to static stimuli and for oscillations unfolding across multiple as compared to only one modality. Stimuli carrying a special appetitive value are also potent synchronizers as are those that seem more responsive (Mills *et al.*, 2019) or human (Kirschner and Tomasello, 2009). We speculate that the benefits associated with these aspects of stimulus complexity are due, in part, to them providing multiple convergent temporal cues that emphasize important rhythmic features. Additionally, they endow objects with perceptual salience, thus facilitating attention capture and stimulus processing. Last, compared to simple stimuli, complex stimuli likely seem more natural and biologically relevant, thereby biasing a positive attitude and a tendency to approach.

## What mechanisms support interactional synchrony?

Humans spontaneously synchronize to a broad range of stimuli including interaction partners. Moreover, in recent years much evidence emerged that social synchronizing is not limited to motor processes but includes the activity of the brain and other bodily systems allowing dyads or groups of individuals to become something like a super-organism. Together, this work has called for attempts to systematically organize evidence and to address the mechanism that enables social synchronization.

The following section provides such an attempt. It first develops a working definition of interactional synchrony. This is followed by a discussion of the key temporal properties that are relevant in co-aligning bodily processes, the (un)importance of wanting to align and the underpinning brain systems. Although our primary interest is in interactional synchrony, we will also review research on how humans align to non-human (e.g. computer-generated) rhythms if that offers relevant theoretical insights.

### Defining interactional synchrony

There are many definitions of interactional synchrony. Some authors adopt a very broad and inclusive perspective by referring to the coordination of biological and behavioral processes during social contact (Feldman, 2017). Others have been more specific and offered a range of conditions that are necessary for an interaction to be called synchronous (Harrist and Waugh, 2002). The one condition that many agree on, and that will be the focus here, is that individuals temporally align with each other (Chetouani *et al.*, 2017).

But what counts as temporal alignment? Are we considering strictly the timing of processes while disregarding their content? If one individual nods and the other concurrently breaths out is that interactional synchrony? What are the relevant temporal parameters (e.g. phase, amplitude) and what level of precision are we looking for? Is temporal alignment an active process in which an oscillator adjusts to another one or is it a passive process that emerges because two oscillators respond to the same stimulus? For example, are individuals with correlated brain activity while watching the same video interactionally synchronizing (Levy *et al.*, 2017; Parkinson *et al.*, 2018)?

Thinking about these questions is important, and although we have only preliminary answers, we will briefly offer them here. First, any bodily process including invisible mental and physiological activity as well as visible behavioral acts can be subject to temporal alignment. Yet, when analyzing such alignment, we should concern ourselves with comparable processes. For example, the dynamics of voluntary muscle movements in one person should be mapped onto voluntary rather than involuntary muscle movements in another person. Second, we consider phase alignment of primary importance and suggest that its precision be scaled to the process of interest. Undoubtedly, alignment errors may be smaller for neuronal as compared with behavioral processes. Last, we see interactional synchrony as an active process in which one or more individuals adjust to each other rather than adjusting independently to stimuli in the environment.

Notably, the thinking outlined here does not consistently map onto the published literature making a review of the mechanisms underpinning interactional synchrony challenging. Thus, in the following review, we may not always be able to strictly adhere to the above principles. However, where possible and relevant, we will highlight potential definitional conflicts.

### Temporal regularity helps us synchronize

Much research attests to the importance of temporal alignment in social interactions (for a review see Schirmer *et al.*, 2016b). An elegant example from the infant literature is work by Nadel and colleagues who positioned infants in front of a monitor that relayed the life recordings from the infant’s mother in another room (Nadel *et al.*, 1999). Intermittently, life interactions were replaced by a playback of good maternal behavior, and this led to a decrease in infant affect. Clearly, timing was important and perhaps as important as the good maternal behavior itself.

Converging evidence from the adult literature comes, among others, from Manera and colleagues who presented two moving agents recorded in the dark with only their joints highlighted (Manera *et al.*, 2013). In two conditions, the agents were either interacting or moving independently. The researchers prepared short stimulus clips in which one side of the recording was obscured by visual noise leaving only one agent clearly visible. They then created two versions of each clip: one in which the obscured agent was present and one in which it was absent. Participants saw both versions of each clip in succession and decided which one contained two agents. Task performance was better when the two agents interacted as compared to when they moved independently. Moreover this effect declined as the researchers introduced a phase delay of 667 ms between the two agents.

A popular approach to understanding how individuals temporally align is to consider their internal and external processes as rhythmical and susceptible to other rhythms in the environment. Indeed this idea has a long theoretical tradition first formalized in Jones’ dynamic attending theory (Jones, 1976; Jones and Boltz, 1989). DAT holds that apart from perceiving time locally, as isolated event durations, we engage in future-oriented timing by representing the relationship of experienced temporal intervals and by creating global temporal structures that enable the prediction of upcoming events.

Following its publication, research has been supporting the tenets of DAT. A paradigm developed by Jones and colleagues (Jones *et al.*, 2002) and adapted by others subsequently (e.g. Escoffier *et al.*, 2010; Miller *et al.*, 2013; Benjamin *et al.*, 2017) entails the presentation of a target stimulus preceded by a task-irrelevant sequence of isochronously spaced stimuli. Targets matching this rhythm elicit more efficient responses than targets occurring slightly earlier or later. Moreover, this holds both when rhythm and target have the same modality (Jones *et al.*, 2002) and when they occur in separate modalities (Escoffier *et al.*, 2010; Benjamin *et al.*, 2017).

A central assumption of DAT is that temporal expectations depend on the metricality of stimulation rhythms. Metricality arises when the intervals of sequential events are hierarchically organized such that there exists an integer ratio of smaller to larger intervals (Jones, 1976; Jones and Boltz, 1989). Metricality is perceived as a rhythmic beat and presents the defining feature of musical rhythms. Additionally, it is believed to characterize interactional rhythms (Jones and Boltz, 1989; Zoefel and VanRullen, 2016; but see Nolan and Jeon, 2014). Yet, current evidence for a role of meter in future-oriented timing and interactional synchrony is limited. Existing work cannot exclude the possibility that rhythmic benefits derive instead from temporal regularity. The use of isochronous sequences (Jones *et al.*, 2002) as well as the repetition of metrical anisochronous sequences (Escoffier *et al.*, 2010) has confounded metricality with regularity (for a more detailed discussion, see Schirmer *et al.*, under review).

Indeed, a large literature highlights regularity as a fundamental feature our brains derive from incoming sensory signals. One part of this literature developed from an initial interest in sensory perception and attention. It employed the mismatch negativity paradigm in which rare deviants occur among frequent standards (Näätänen, 1990, 1995). Participants are distracted from these stimuli with a book or a silent movie, and their electroencephalogram (EEG) is recorded. The event-related potential (ERP) derived from the EEG shows a negative deflection signaling that deviant sounds recruit more processing resources than standard sounds above and beyond what would be expected due to sensory habituation. Importantly, this is true no matter how deviants and standards differ. Effects emerge for a range of features such as pitch, intensity, spectral properties and, of course, time (Näätänen *et al.*, 1989; Schirmer *et al.*, 2016a). They show also for fairly complex input that combines two acoustically overlapping sequences with the deviant being irregular in only one of them (Rahne *et al.*, 2007; Bendixen *et al.*, 2012).

Evidence for the importance of regularity also comes from research on language learning. Interested in how infants identify words in continuous speech, Saffran and colleagues examined a potential role for the transition probabilities of syllable pairs unfolding within (*P* = 1) and between (*P* = 0.33) four three-syllable nonsense words (Saffran *et al.*, 1996). The researchers noted that 8-month-old infants use what they called ‘statistical learning’ to achieve word segmentation within only 2 min. Again, this original result has been replicated across different senses, for more complex stimuli and across age groups (for a review, see Fiser and Lengyel, 2019). Moreover, it was linked to the mismatch negativity, which can be evoked by the violation of transition probabilities in an auditory sequence (Mittag *et al.*, 2016).

**Fig. 1:**
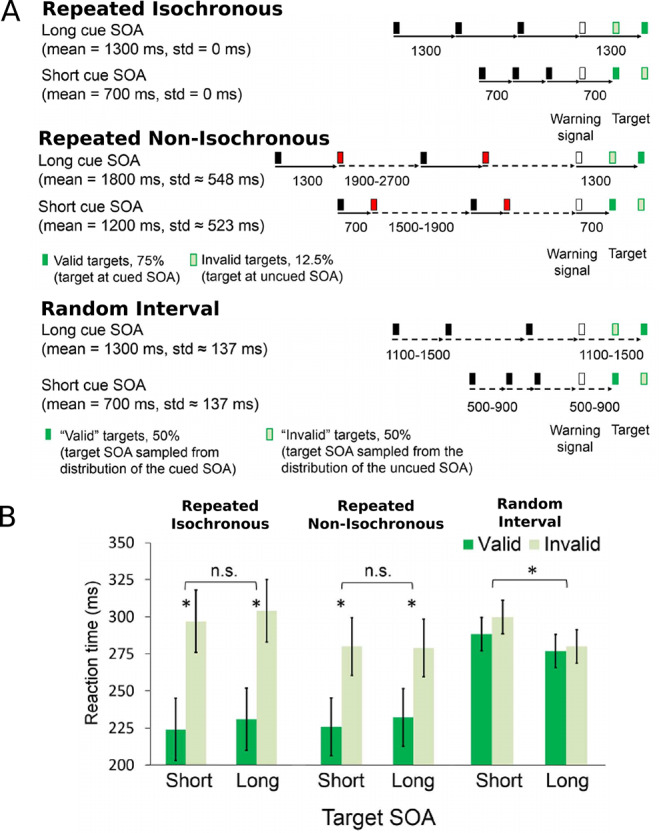
Breska and Deouell (2017) stimuli and results. (A) Subjects detected targets embedded in a stream of visual stimuli. In the repeated isochronous condition, the stream of intervals was fixed. In the repeated non-isochronous condition, every black-to-red interval was fixed and red-to-black intervals were jittered. In the random interval condition, all intervals were jittered around the fixed interval. In the first two conditions, the target (dark green) appeared at the fixed SOA relative to a warning signal (white) in 75% of the trials and at the other SOA in 12.5% of the trials (light green with dark green edge). The remaining 12.5% were catch trials in which no target appeared, to prevent anticipatory responses to long SOA targets. In the random interval condition, the target SOA was drawn from the same distribution as the stream SOA in 43.75% of the trials and from the other distribution in the other 43.75%; again, 12.5% of the trials were catch trials. (B) Mean reaction times for each combination of SOA and cue validity in the three experimental conditions. Error bars represent standard errors of the validity effect within each SOA and condition. ^*^P < 0.05.

To date, few attempts have been made to pursue temporal regularity in the context of rhythm perception or interactional synchrony. One step in this direction is work by Breska and colleagues who presented a defined temporal interval either rhythmically as a continuous repetition or non-rhythmically by introducing variable delays between interval repetitions (Breska and Deouell, 2017). After each sequence, a warning stimulus appeared followed by a target stimulus with either the defined temporal interval or another interval (Figure 1). Compared with a random interval stimulus sequence, both defined interval conditions produced the same benefits on behavioral responses and EEG oscillations. Indeed, the only special effect of the metrical isochronous rhythm was on motor preparatory brain activity, suggesting that meter or periodicity may be specifically important for motion planning. Yet, in terms of ongoing mental processing and behavioral responding, regularity was the more critical factor.

Convergent results come from work by Schirmer and colleagues who made a first attempt at manipulating metricality and regularity orthogonally (Schirmer *et al.*, under review). They presented task-irrelevant sequences composed of musical measures with high or low metricality and either repeated or randomly varied measures throughout a sequence. Visual target processing was independent of measure metricality and target timing. On-beat targets were not processed beneficially relative of off-beat targets in highly metrical sequences. However, measure repetition facilitated visual attention as reflected in behavioral and EEG/ERP responses. Thus, Schirmer and colleagues argue that regularity trumps metricality in its relevance for temporal alignment. Indeed, given the dynamical nature of social interactions (Issartel *et al.*, 2015; Dahan *et al.*, 2016; Mills *et al.*, 2019; Washburn *et al.*, 2019), behavioral periodicities are often transient and may be more readily represented on short, interval-based scales than on global metrical ones.

### Intentionality facilitates interactional synchrony

Both the mismatch negativity and statistical learning suggest that we represent (temporal) regularity more or less automatically. Our brains detect change in stimulus sequences that are currently unattended. For simple physical changes, they can do this early in development, as evidenced by EEG recordings done on fetuses (Draganova *et al.*, 2005), and while we sleep (Strauss *et al.*, 2015). Similarly, statistical learning—a process that precedes change detection—operates fast and without intention although it appears to require wakefulness (Farthouat *et al.*, 2018). Thus, one may speculate that also interactional synchrony emerges from temporal regularity without intention.

Yet, several studies on interactional synchrony seem to contradict this notion. As reviewed above, music studies identified a role of the music-making partner, and this was in part driven by the partner’s behavior and in part by the participant’s attitude (Kirschner and Tomasello, 2009; Zhang *et al.*, 2016; Mills *et al.*, 2019). The latter, attitudinal effect is also supported by research on non-musical social interactions (Kinreich *et al.*, 2017). For example, the temporal alignment of motor and physiological arousal depends on feelings toward interaction partners and social rapport (for a review, see Schirmer *et al.*, 2016b). We synchronize more readily with friends than strangers, and with strangers we find likable as compared to unlikable.

Evidence comes from research on spectators and active participants of an arousing ritual where at one point in the ritual the active participants walk across a carpet of red-glowing coal. The closeness of the relationship between spectators and active participants predicts their heart rate synchrony during the ritual (Konvalinka *et al.*, 2011). Additionally, a role for likability was demonstrated in a synchronous stepping task where participants more readily aligned their motion with another ostensible participant who came to the experiment on time as compared to late (Miles *et al.*, 2010). Last, a study employing the trust game, popular in the decision-making literature, suggests we synchronize selectively. In this game, one participant is given an endowment she/he is free to invest. Investments are made by transferring some or all of the endowment to a second participant. What this second participant receives is automatically tripled, and she/he may decide what if anything to return to the first participant. Playing this game induces greater neuronal synchrony between participants when it is framed as a power instead of a trust game (Sun *et al.*, 2019).

Taken together, available evidence indicates that we need very limited resources to represent temporal regularity in the environment. Moreover, we can track, unintentionally, not just one but multiple stimulus timelines. In contrast, we adjust our own temporal rhythms to external ones in a more selective manner. Positive interactions more readily elicit synchrony than neutral or negative ones. Yet, whether these effects emerge in a controlled, effortful manner is questionable. Indeed some findings suggest that they don’t. When playing ‘Whac-a-Mole’, synchrony between players emerges gradually as a function of player visibility despite the fact that such synchrony impairs individual performance (Naber *et al.*, 2013). Thus, we speculate that selectivity in our propensity to synchronize is coupled to socio-emotional processes that may operate outside awareness. They serve as a filter that prevents us from synchronizing randomly in ways that may be detrimental and directs us to important oscillators for which synchronizing may be beneficial.

### How does the brain help us synchronize?

Now that we have established a dissociation between passively representing any external temporal regularity and selectively synchronizing with it, we consider how both types of processes are implemented in the brain. In light of extant work, it seems that the representation of temporal regularities is supported by modality- and timing-specific regions, whereas the motivation to align with these regularities is driven by a socio-emotional processing network.

The brain representations of temporal regularity have been pursued from a process perspective leveraging on the high temporal resolution of the EEG and from a structural perspective with lesion patients and magnetic resonance imaging (MRI). EEG research has focused on oscillations in post-synaptic activity and on how their temporal properties change as a function of stimulus timing. The best replicated finding is that exposure to a regular stimulus stream amplifies the stimulation frequency in the EEG over relevant sensory regions—a phenomenon called frequency tagging (for a review, see Wieser *et al.*, 2016). For example, a visual stimulus rhythm of 20 Hz amplifies 20 Hz oscillations as well as associated harmonics in the occipital EEG. Such amplification emerges in a bottom-up manner based on stimulus characteristics as well as in a top-down manner based on perceptual processes and attention. Indeed, imagining a particular metrical beat can enhance corresponding EEG frequencies (Nozaradan *et al.*, 2011).

EEG oscillations are characterized not only by their power. They can also be quantified in terms of their phase and how this phase aligns with, for example, the onset of a stimulus or the phase of oscillations measured at other channels or a different head. Many studies have pursued whether and how the presence of an external oscillator changes the phase of EEG oscillations. Yet, reviewing this literature is challenging as different investigators focused on different frequency bands and phase measures (e.g. Myers *et al.*, 2014; Escoffier *et al.*, 2015; Zoefel and VanRullen, 2016; Breska and Deouell, 2017; Herbst and Obleser, 2019). There appears to be some convergence of findings for lower frequencies in the theta and delta band (for reviews, see Rimmele *et al.*, 2018a, 2018b). However, whether they reflect changes in phase alignment or a coordinated phase reset due to sensory processes and associated sensory expectations requires further and better concerted efforts.

Research aimed at identifying the brain structures representing temporal information and regularity supports a role for modality-specific systems and additionally highlights a central timer (Coull *et al.*, 2011; Merchant *et al.*, 2013). Many now assume that the striatum monitors and integrates temporal information represented by the cortex. A popular framework, the striatal beat-frequency model, proposes a role for dopamine signaling in synchronizing cortical activity at the onset of a stimulus and in tracking ensuing phase patterns as markers for the passage of time (Buhusi and Meck, 2005). Interestingly, similar proposals emerge from the literature on the statistical learning of non-temporal transition probabilities (Bach and Dolan, 2012; Dehaene *et al.*, 2015; Davis and Hasson, 2018), suggesting a perhaps more general role of striato-cortical loops in our ability to derive temporal and other kinds of regularity from the environment. Anatomical overlap with reward-related circuitry likely supports the apparent coupling between striatal regularity processing and positive affective experiences (Launay *et al.*, 2016).

More recently, efforts increased to pin down how brains leverage temporal representations to synchronize the mental and behavioral activity across individuals. Research focusing on EEG oscillations has highlighted a role for increased power in the gamma band (Kinreich *et al.*, 2017; Pratt *et al.*, 2018). For example, mother–child dyads watching a video of a past joint interaction show a correlated increase in gamma power for sections in which there is high but not low behavioral synchrony (Levy *et al.*, 2017). Do note, however, that it remains to be determined whether these and similar effects are simply due to joint attention being greater for sections more relevant to both individuals such that brain coupling arises from enhanced attention to the same physical stimulus.

Again, findings are less consistent for EEG oscillatory phase. Different researchers have reported effects for different frequency ranges including the alpha (Goldstein *et al.*, 2018), beta (Novembre *et al.*, 2017) and theta band (Leong *et al.*, 2017) as well as for different phase measures including phase coupling between individuals (Novembre *et al.*, 2017; Goldstein *et al.*, 2018), partial directed coherence (Astolfi *et al.*, 2015), general partial directed coherence (Leong *et al.*, 2017) or total interdependence (Dikker *et al.*, 2017).

Importantly, there appears to be convergence as regards the relevant brain structures. Attempts to localize synchronizing effects in the EEG (Kinreich *et al.*, 2017; Levy *et al.*, 2017; Pratt *et al.*, 2018) as well as work relying on measures of blood oxygenation (Jiang *et al.*, 2015; Atzil *et al.*, 2017) point to the posterior superior temporal cortex. For example, synchronization of gamma power in the context of social interactions can be traced back to dipoles in the temporo-parietal junction (Kinreich *et al.*, 2017; Levy *et al.*, 2017; Pratt *et al.*, 2018). Speaker–listener fMRI signal coupling was shown to be higher for verbal content with high as compared to low predictability, and this effect peaked in the posterior STG (Dikker *et al.*, 2014). Additionally, in groups of three with one individual emerging as the leader, blood oxygenation as measured with near infrared spectroscopy correlated in the TPJ for leader–follower but not follower–follower pairs (Jiang *et al.*, 2015). Together, these results align with evidence that the posterior STS/TPJ represents an important hub for the multimodal representation of social signals (Schirmer and Adolphs, 2017) and that it contributes to socially relevant temporal computations (Schirmer *et al.*, 2016b).

### Putting things together

**Fig. 2:**
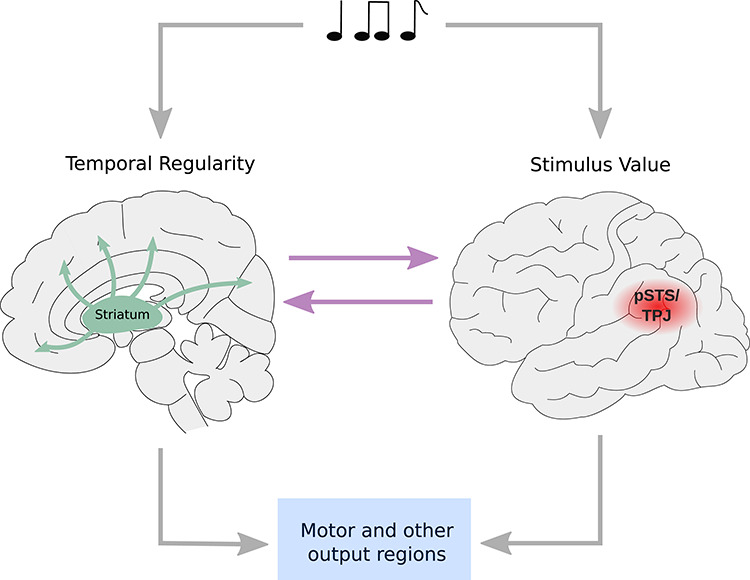
Proposed theoretical framework for the mechanisms underpinning interactional synchrony.

A bird’s-eye perspective of the evidence reviewed here suggests a potential theoretical framework for directing future research on the processes underpinning interactional synchrony (Figure 2). Specifically, it highlights temporal regularity—irrespective of periodicity or metricality—as a fundamental cue humans derive without intention from their environment. In social interactions, our species automatically represents the durations of another’s behavioral expressions (e.g. nodding, arm gestures, speech). Moreover, if these durations are regular, they may be actively utilized to align one’s own mental and behavioral processes. Whether alignment occurs depends on a range of factors that prompt an individual’s social interest and desire to interact with the other. At the level of the brain, temporal perception and alignment value can be functionally and structurally dissociated. Temporal perception is enabled by cortical neuron assemblies and the integration of their signals by striato-cortical loops acting as a central timer or perhaps more generally as a regularity detector. Alignment value depends on socio-emotional computations and informs temporal processes via communication between the temporal and the social brain. Specifically, a signal with temporal regularity may recruit posterior superior temporal cortex for socio-emotional weighting. Possibly, the computations initiated there feedback to internal clock mechanisms as well as to output systems in the frontal lobe that then adjust the temporal course of both mental and behavioral processes.

## What are the benefits of interpersonal synchrony?

Interpersonal synchrony is an almost ubiquitous characteristic of human social interactions and one of the reasons why social psychology and neuroscience moved from focusing on an individual participant to studying dyads or groups (Schilbach *et al.*, 2013; Redcay and Schilbach, 2019). Although behavioral synchrony often arises unintentionally (Schmidt and O’Brien, 1997; Richardson *et al.*, 2005, 2007; Issartel *et al.*, 2007), people also actively create synchrony when, for example, performing music or dancing together. Our proclivity to align behavior and neurophysiological processes depends on the relationship we have (Konvalinka *et al.*, 2011; Reindl *et al.*, 2018) and the social rapport we experience (Miles *et al.*, 2010). Conversely, experimentally inducing motor synchrony enhances rapport and prosocial behavior in children (Cirelli *et al.*, 2014) as well as adults (Kokal *et al.*, 2011). Thus, we can assume that social rapport and interpersonal synchrony bidirectionally reinforce each other acting as a ‘social glue’ that binds individuals together (Lakin *et al.*, 2003; Keller *et al.*, 2014).

In this section, we focus on the question of how interpersonal synchrony benefits interactions. We begin by outlining the influence of interpersonal synchrony during face-to-face exchanges on communication outcomes. In particular, we suggest that synchrony might be useful in terms of facilitating prediction, thereby enhancing the processing of shared information. We will then discuss the potential affiliative benefits of interpersonal synchrony, especially when building relationships based on mutual commitment and secure attachment. The section will close with an attempted broader perspective that considers other potential benefits and their situation within the course of human evolution.

### Synchrony facilitates prediction

Navigating complex dynamic environments is a vital challenge for all living organisms. Humans have evolved the extraordinary ability to coordinate within large groups of individuals (Brothers, 1990; Herrmann *et al.*, 2007; Launay *et al.*, 2016). According to predictive processing accounts, brains are constantly engaged in a process of optimizing internal models of the external world as well as the organism itself (Friston, 2005). As sensory information is noisy and offers only incomplete information, it is essential that inferences based on sensory inputs are continually improved and internal predictive models are optimized in order to reduce prediction error (Friston, 2010). In social interactions, this might involve predicting another person’s motor commands given prior expectations of the person’s goal and then, given the expected motor commands, predicting specific action kinematics (Kilner *et al.*, 2007; Schütz-Bosbach and Prinz, 2007). According to this view, predicted kinematics are then compared to observed kinematics. Mismatches between both generate prediction errors, which are used to optimize further predictions. On the basic level of motor commands and action kinematics, it seems plausible that behavioral synchrony within dyads and groups might render the interacting partners’ actions more predictable for each other, thus reducing prediction error.

### Communicative benefits

Miles *et al.* (2009) argue that interactional synchrony reduces working memory load and facilitates information flow because freed-up cognitive resources can be directed toward the perception of the other person. Indeed, incidental memory for an interaction partner was found to be enhanced following synchronized movement (Macrae *et al.*, 2008). The notion that synchrony supports communication has been put forth and tested predominantly in the domain of verbal conversations (Hasson *et al.*, 2012), although mutual adaptation of neural activity has also been reported in gestural communication (Dumas *et al.*, 2010; Schippers *et al.*, 2010). The speech signal contains amplitude modulations at specific rhythms, notably temporal regularities in syllabic and word boundaries, corresponding in their frequency range to the EEG theta rhythm (Luo and Poeppel, 2007). Especially in a noisy environment, selectively entraining one’s neuronal processes to the speech envelope of a speaker offers a considerable processing advantage as phases of high neuronal excitability in the listener can be timed to co-occur with the incoming speech input (Obleser and Kayser, 2019). For instance, Zion Golumbic *et al.* (2013) showed that a listener’s brain activity dynamically tracks an attended speech stream, thus amplifying the signal and increasing signal-to-noise ratio. Interpersonal neural synchrony (or ‘brain-to-brain coupling’) has therefore been described as a process coupling the sensory system of a listener to the motor system of a speaker (Hasson *et al.*, 2012). This process might explain why interpersonal neural synchrony has consistently been linked with enhanced mutual understanding (Kuhlen *et al.*, 2012).

Importantly, during live exchanges, this process will likely be reciprocal and benefit from mutual adjustments of each partner to the other. This is nicely demonstrated in caregiver–infant interactions, where contingent responses of the caregiver induce more mature vocalizations of the infant (Goldstein *et al.*, 2018), while caregivers adjust to their infants by producing more simplified speech in response to the infants’ babbling (Elmlinger *et al.*, 2019). Correspondingly, for preschoolers and their caregivers, the degree of neural synchrony during a face-to-face interaction is positively related to dyadic behavioral reciprocity (Nguyen *et al.*, 2020) and conversational turn-taking (Nguyen *et al.*, n.d.). Together, these results speak to the notion of interactional synchrony as a bidirectionally adaptive process in social exchanges.

In addition to the speech signal itself, visual cues linked to speech as well as speech-accompanying gestures might support this process. For instance, rhythmic or temporally regular mouth, head and hand movements, which are coupled to the auditory speech signal, might help the listener to adjust brain activity to the speech input (Hasson *et al.*, 2012). This idea is in line with EEG evidence that rhythmic or temporally regular stimuli presented in one modality adjust oscillatory processes in another modality (Escoffier *et al.*, 2015; Schirmer *et al.*, under review). Furthermore, mutual eye contact has been suggested to induce a simultaneous phase reset in both communicating partners’ neural oscillations (Jiang *et al.*, 2012; Leong *et al.*, 2017). This could facilitate interpersonal neural synchronization in face-to-face interactions.

Whereas research on interpersonal synchrony of brain dynamics has focused on verbal communication, synchronization of autonomous physiological activity is mostly investigated in the context of emotional aspects of interpersonal exchanges, including affect sharing and emotional co-regulation. For instance, a range of studies has linked caregivers’ and infants’ behavioral and affective attunement with interpersonal synchrony of physiological parameters (Leclere *et al.*, 2014), such as respiratory sinus arrhythmia (Feldman *et al.*, 2011), hormonal activity (Feldman, 2017) and thermal facial imprints (Ebisch *et al.*, 2012), among others. As further discussed in the section on long-term affiliative benefits of synchrony below, sharing of affect between infant and caregiver is thought to play a vital role for building first affective bonds (Feldman, 2017; Busuito *et al.*, 2019). Mutual attunement of physiological activity is also discussed as critically facilitating interpersonal sharing of affect between adults (Konvalinka *et al.*, 2011), sometimes mediated through affective touch (Goldstein *et al.*, 2018; Wijaya *et al.*, 2019).

It is important to note that while basic forms of affect sharing may well benefit from a high level of interpersonal physiological synchrony, in situations involving a high level of negative arousal, physiological synchrony might result in empathetic distress rather than empathetic concern. Distress facilitates egoistic motivations (e.g. to leave the situation) rather than prosocial behavior focused on helping another person (Decety and Meyer, 2008; Lamm *et al.*, 2016). More specifically, for other-directed empathetic concern to arise, there needs to be a clear self-other distinction as well as functional emotion regulation (e.g. cognitive appraisal) (Decety and Meyer, 2008; Lamm *et al.*, 2016). A high degree of affective synchrony and self-other overlap, in contrast, may be detrimental to higher-level controlled affective processes such as emotional perspective-taking and empathetic concern. Supporting this notion, a recent study linked increased behavioral synchrony in whole-body movements to decreased affective self-regulation (Galbusera *et al.*, 2019).

### Affiliative benefits

We have focused thus far on the proximate effects of interpersonal synchrony in face-to-face interactions. Considerably fewer studies have addressed the question of whether and how interpersonal synchrony may relate to longer-term outcomes of social exchanges, such as relationship quality. There is some evidence suggesting bidirectional links between moment-to-moment interpersonal synchrony and long-term characteristics of relationships. Several studies found that the degree of neural and physiological synchrony achieved during an interaction is influenced by the pre-existing relationship between the interaction partners (Konvalinka *et al.*, 2011; Pan *et al.*, 2017; Reindl *et al.*, 2018).

For instance, in a collaborative task, 5- to 9-year-old children synchronized brain activities with their mother, but not with an unfamiliar female (Reindl *et al.*, 2018). Thus, on the one hand, having a close relationship with another person seems to facilitate proximate interpersonal synchrony. On the other hand, both interpersonal neural synchrony and synchrony of finger movements were found to increase between previously unfamiliar individuals following a collaborative task (Yun *et al.*, 2012). Even within a short time period, getting to know each other seems to facilitate interpersonal synchronization. Thus, there may be a bidirectional relationship between one’s attitude toward an interaction partner and one’s readiness to synchronize. Both likely influence each other.

Indeed, behavioral synchrony can be quite easily induced in dyads and groups and may be used to increase mutual liking, rapport and perhaps even group cohesion. Experimental research has consistently demonstrated that behavioral synchrony increases helping and prosocial sharing in infants (Cirelli *et al.*, 2014; Trainor and Cirelli, 2015), children (Rabinowitch and Meltzoff, 2017) and adults (Kokal *et al.*, 2011). For instance, 14-month-olds who were bounced in synchrony with an adult experimenter showed increased instrumental helping behavior toward the bouncing partner and also the partner’s affiliate, but not toward another neutral adult (Trainor and Cirelli, 2015). Similarly, preschoolers act more prosocially toward peers following a synchronous movement game (Rabinowitch and Meltzoff, 2017). A recent study in adults showed that moving in synchrony with each other promoted self-reported rapport, though not learning *per se*, during a teacher–learner interaction (Nozawa *et al.*, 2019). Given that by 12 months of age, infants prefer synchronous over asynchronous social partners (Tuncgenc *et al.*, 2015), it seems that infants already evaluate people based on their behavioral alignment with them. Behavioral synchrony may be an informative clue for individuals to identify ‘good’ social partners with whom they are consequently more ready to engage in reciprocal exchanges.

An impressive body of research has linked behavioral and physiological attunement between infants and their caregivers, especially their mother, to the formation of secure attachments (Feldman, 2017). Given that infants are highly dependent on their caregivers to maintain physiological homeostasis (Fotopoulou and Tsakiris, 2017), it makes sense to assume that a highly sensitive and attuned caregiver is better able to meet the infant’s needs in any given moment and thus to establish a secure attachment in the long run. Interestingly, however, a number of empirical findings support an ‘optimum midrange model’ of contingency in caregiver–infant interactions (Beebe *et al.*, 2008; Beebe and Steele, 2013). Both disengaged parenting and too intrusive parenting and overstimulation have been associated with insecure attachment outcomes. In addition, there is evidence for a dissociation between behavioral coordination and aspects of physiological alignment. In particular, correlations of cortisol levels were higher in parent–child dyads with less behavioral coordination (Saxbe *et al.*, 2017). In line with the notion that too much physiological synchrony in caregiver–child interaction might be detrimental, Wass *et al.* (2019) report greater co-fluctuation of arousal throughout the day in caregiver–infant dyads with an anxious caregiver. Whereas non-anxious parents responded with arousal primarily to peaks in their infant’s arousal, anxious parents responded also to small fluctuations of their infant’s arousal. Excessive physiological responses to infants’ expressions of distress were also reported in physical child abusers and individuals at risk for being physically abusive (McCanne and Hagstrom, 1996).

### A broader perspective

The distinction between the two benefits discussed thus far is admittedly somewhat artificial. Naturally, one might presume a reciprocal relationship between communication and affiliation in the sense that an optimal exchange of ideas facilitates bonding and vice versa, thus blurring a specific role of synchrony on either. Moreover, there are likely other and perhaps more primal benefits to synchronizing. For example, some proposed an original function in courtship displays. Research suggests that such displays are more powerful, noticeable and informative to potential mates if enacted as a group (Merker *et al.*, 2009). Yet, other activities of our ancestors and fellow humans today benefit from temporal coordination. This includes things like hunting, gathering and fighting as well as skilled manual labor, team sport or music-making (Mithen, 2005). In fact, some argue for a role of synchronizing in our ability to act and live in groups that far outnumber those of our primate relatives. Synchronizing is seen as a means to fostering group cohesion that in part replaced the more laborious and time-consuming grooming (Launay *et al.*, 2016). Thus, at different levels ranging from the dyad to a large group and from very specific tasks to more general social dynamics, synchronizing affords a broad range of benefits that have been and still are drivers for its frequent occurrence when humans come together.

## Conclusions

The last couple of decades have seen a significant increase in synchrony research. Whether and how we synchronize has been approached from a cognitive, social, developmental, biological and evolutionary perspective. While these perspectives are slowly merging, we are still without a holistic understanding of the underlying processes. To push matters further, we here reviewed the field with an interest in the stimuli, mechanisms and benefits of interactional synchrony.

Looking at the role of stimulus characteristics, we observed an advantage for more complex stimuli. An additional modality or emotional meaning and some amount of variability appear relevant in prompting synchronization—perhaps because they make a stimulus seem more natural and meaningful.

Looking at mechanisms, we found that traditional views on the importance of a beat-based structure are not supported by more recent data highlighting the representation of temporal regularity as a more fundamental process. Indeed, periodicity, meter and musical rhythm may be best understood as special instances of regularity that are not strictly necessary for us to temporally align. Although we represent temporal regularity fairly automatically and may adapt to such regularity without intention, whether or how closely we adapt depends on a rhythm’s perceived socio-emotional significance. Internal timing processes implemented by striato-cortical loops and evaluative processes dependent on the pSTS/TPJ support these two synchronizing stages.

Together, insights into the stimulus properties and mechanisms converge with evidence that interactional synchrony confers a range of social benefits. Recognizing and aligning with temporal regularity makes the other(s) more predictable which then facilitates joint attention and action, information exchange and the formation of affective bonds. Because synchronizing works in dyads as well as in crowds, it may serve as a social adhesive that engenders affection for others and feelings of belonging.

## Funding

This work was supported by a GRF grant awarded by the Hong Kong Research Grants Council to Annett Schirmer (14612318).
